# Psychiatry Trainees’ Views of Relational Prescribing: A Mixed Methods Scoping Activity

**DOI:** 10.1192/bjo.2025.10799

**Published:** 2025-06-20

**Authors:** Haroula Konstantinidou, Joanna Male, Lauren Turner, Dolly Sud

**Affiliations:** Leicestershire Partnership NHS Trust, Leicester, United Kingdom

## Abstract

**Aims:** Medicine-taking is a complex human behaviour. Psychiatry is returning to the Bio-Psycho-Social paradigm with a re-emphasis on the relational aspects of prescribing. To improve knowledge/understanding of the key concepts of Relational Prescribing, three one-hour seminars each one week apart were delivered to clinicians at Leicestershire Partnership NHS Trust (September 2022). The overall aim was to understand the views of psychiatry trainees.

**Methods:** Seminars, delivered by a Senior Psychiatric Pharmacist and a Consultant Psychiatrist in Psychotherapy, included evidence and core concepts of relational prescribing.

Quantitative data was collected by pre- and post-seminar surveys consisting of Likert style questions. Ordinal analysis was applied, and findings reported as descriptive statistics.

A scoping activity consisting of an online group discussion using a semi-structured topic guide was used to collect qualitative data to get a more in-depth understanding. This was recorded, transcribed verbatim, anonymised and analysed using Framework Analysis (a type of Thematic Analysis) – chosen because it emphasises both a priori issues and themes identified.

**Results:** Forty-seven participants completed a pre-seminar survey, thirteen returning both pre- and post.

Six participants joined the online discussion (March 2023, lasting one hour and fifty-five minutes). Four themes were identified.

Clinician factors. Professional identity as “the prescriber”, the clinician’s conceptualisation of medication-as-object, and experience and confidence of the clinician.

Patient factors. The patient’s expectations and the patient’s experience of, and meaning attached to, medication.

Consultation factors. “Gentle dialogue” and the doctor-as-the-drug, and consultation dynamics.

Context factors. Clinical setting, brevity of appointments, lack of continuity, and service dynamics.

**Conclusion:** Quantitative data indicated increased confidence in Relational Prescribing (although not statistically significant). Trainees felt the most confident exploring experiences of and attitudes towards medication-taking, with less confidence in psychoeducation and patient ambivalence. Prescribing is a relational exchange involving action. Action provides relief for both parties, meeting the patient’s expectations for a concrete response to their distress and the clinician’s expectation of a powerful and impactful professional identity. Prescribing was often felt the only way to hold the anxiety. Internal and external pressures to prescribe were considered. Clinicians described the multi-layered meaning of a prescription, from the offer of an apology to an exertion of power. Clinicians felt trapped by lack of seniority and confidence. The rotational nature of training, brevity of appointments, and lack of continuity of care were felt to hinder a relational approach. The phrase “gentle dialogue” emerged de novo suggesting that non-pharmacological interventions may be felt less valid.

